# How do glucagon‐like Peptide‐1 receptor agonists affect measures of muscle mass in individuals with, and without, type 2 diabetes: A systematic review and meta‐analysis

**DOI:** 10.1111/obr.13916

**Published:** 2025-04-03

**Authors:** Oluwaseun Anyiam, Arash Ardavani, Rushdina Sofia Abdul Rashid, Avinash Panesar, Iskandar Idris

**Affiliations:** ^1^ MRC/ARUK Centre for Musculoskeletal Ageing Research and National Institute for Health Research (NIHR), Nottingham Biomedical Research Centre (BRC), School of Medicine University of Nottingham Derby UK; ^2^ Department of Endocrinology and Diabetes University Hospitals Derby and Burton NHS Foundation Trust Derby UK; ^3^ Department of Endocrinology and Diabetes United Lincolnshire Hospitals NHS Trust Lincoln UK; ^4^ Derbyshire Healthcare NHS Foundation Trust Derby UK

**Keywords:** glucagon‐like peptide‐1 receptor agonist, muscle mass, obesity, type 2 diabetes

## Abstract

Glucagon‐like peptide‐1 receptor agonists (GLP1RAs) are used for the management of type 2 diabetes (T2DM) and obesity. GLP1RAs induce significant weight loss but concerns have been raised regarding the associated effects on muscle mass (MM). We therefore conducted a systematic review and meta‐analysis assessing the effects of GLP1RAs on various measures of MM in individuals living with overweight or obesity, with and without T2DM. Comprehensive search of Medline, Pubmed, EMBASE, CINAHL, the Cochrane Central Register of Controlled Trials (CENTRAL), and Google Scholar was performed. Studies involving cohorts with a mean age over 40 years and a mean body mass index over 25 kg.m^‐2^ were included. The primary outcome was any measure used to estimate MM, whilst fat mass (FM) and total body weight were included as secondary outcomes. Thirty‐eight publications, involving 1735 participants, were included in the review. Separate meta‐analyses were performed for studies involving participants with T2DM and individuals without T2DM (non‐DM). In individuals with T2DM, GLP1RAs induced a non‐significant mean reduction in MM measures (‐0.74 kg, 95% CI: ‐1.61, 0.14, p = 0.10), despite significantly reducing FM (‐3.18 kg, 95% CI: ‐4.09, ‐2.28, p < 0.0001). In the non‐DM analysis, a significant mean reduction in MM measures was observed (‐1.41 kg, 95% CI: ‐2.12, ‐0.71, p = 0.0001), however, this was significantly less than the reduction in FM (‐6.02 kg, 95% CI: ‐7.53, ‐4.50, p < 0.0001). In both populations, the reduction in measures of MM accounted for less than 20% of the total weight reduction. These findings provide some clarity to clinicians that use GLP1RAs to manage individuals with T2DM and/or obesity, however, further more detailed analysis of the impact of these medications on functional skeletal muscle is required.

## 1 INTRODUCTION

Type 2 diabetes (T2DM) affects over 500 million adults worldwide, and this figure is projected to rise to over 750 million by 2045.^1^ The association between T2DM and excess weight is well described, approximately 85–90% of individuals with T2DM are classified as either overweight or obese.^2^ Global rates of overweight and obesity are also rising,^3^ inevitably contributing to this trend in T2DM prevalence. Weight loss is widely recognized to lead to improved glycaemic control,^4^ and reduced incidence of complications.^5^, ^6^ Consequently, interventions that improve glycaemic control, whilst also inducing significant weight loss, are highly sought after.^7^

Glucagon‐like peptide 1 receptor agonists (GLP1RAs) are a class of medications that are widely used for the management of T2DM. Amelioration of hyperglycemia occurs via stimulation of insulin secretion and suppression of glucagon release from pancreatic cells,^8^ along with improved beta cell function.^9^, ^10^ In addition, GLP1RAs act on enteric receptors to slow gastric emptying, as well as centrally to modulate satiety centers in the human brain,^11^ the latter inducing reductions in appetite and food intake, resulting in weight loss.^12^ This constellation of effects has made GLP1RAs highly attractive interventions for the management of T2DM, and currently a range of medications in this class are indicated for treating T2DM with concurrent obesity.^13^ More recently, the powerful weight‐reducing effects of GLP1RAs have been exploited for use in the management of obesity. Following the results of the SCALE^14^, ^15^, ^16^ and STEP^17^, ^18^, ^19^, ^20^ series of studies, two GLP1RAs – Liraglutide and Semaglutide – have been approved for weight management in individuals living with obesity.

An important aspect of weight loss through GLP1RAs and other weight loss interventions, however, is the associated significant reduction in measures of muscle mass (MM). Reductions in measures such as lean body mass (LBM) and fat‐free mass (FFM) have been reported to be as high as 40% of the total amount of weight lost.^21^, ^22^ Weight regain, however is thought to almost entirely consist of fat.^23^ Recurrent cycles of weight loss and weight regain leads to body composition changes that contribute to ongoing difficulties with maintaining weight loss, putting affected individuals at an increased risk of sarcopenic obesity and long‐term adverse metabolic health.^23^ Skeletal muscle is the body's largest metabolically active tissue, contributing to approximately 50% of whole‐body protein turnover^24^ and 70% of post‐prandial glucose disposal.^25^ Lower levels of MM have been robustly linked to higher insulin resistance,^26^ sarcopenia, and frailty,^27^ and is an independent predictor of morbidity and mortality risk.^28^, ^29^ Conservation of muscle mass and function across the life‐course is therefore imperative for the maintenance of healthy aging.

MM naturally increases throughout childhood and adolescence, peaking between the ages of 30 – 40 years, before gradually declining thereafter.^30^, ^31^, ^32^ Notably, this natural loss of MM during later life is accelerated in individuals with T2DM compared to those without T2DM,^33^ leading to a higher incidence of frailty among people living with this condition.^34^ Furthermore, obesity itself is strongly associated with poor muscle quality and function.^35^ To that end, the preservation of MM is of utmost importance in individuals living with obesity, particularly those with concurrent T2DM, and must be given due consideration when choosing interventions that promote weight loss in these populations.^22^, ^35^

Several studies have assessed the impact of GLP1RAs on measures of MM, with varied results. One review highlights this, reporting 4–60% of total weight loss arising from LBM.^36^ Another more recent review suggests GLP1RA treatment may have a protective effect on MM in individuals with T2DM.^37^ Intriguingly, a number of epidemiological and pre‐clinical studies, including from our group, have reported positive benefits of GLP‐1 on various markers of muscle mass and metabolism.^38^, ^39^, ^40^

However, the impact of GLP1RAs on measures of MM in the context of clinical practice remains unclear. Specifically, no quantitative examination of the effect of GLP1RA‐induced weight loss on measures of MM has previously been performed. Furthermore, no assessment of the differential muscle effects of GLP1RAs in individuals treated for T2DM, and those treated for obesity without T2DM, has occurred, despite GLP1RAs being recommended for use in both conditions. Finally, no previous review has related these effects to changes in weight and fat mass, which are also important considerations when using these medications. Thus, this systematic review and meta‐analysis were performed to fill these gaps in the literature by assessing the effect of GLP1RAs on measures of MM, relative to changes in fat mass (FM) and total body weight (TBW) in individuals with, and without T2DM.

## 2 METHODS

This systematic review was conducted in accordance with the 2020 Preferred Reporting Items for Systematic Reviews and Meta‐Analysis (PRISMA) guidance, and a PRISMA checklist is provided in the supplementary material (Appendix S1). The review protocol is registered in the International prospective register of systematic reviews (PROSPERO 2022), registration number CRD42023489617. The full review protocol is located in Appendix S2 of the supplementary material.

The overall aim of the review was to examine absolute changes in measures of MM among individuals with overweight, with and without concurrent T2DM, relative to corresponding alterations in FM and TBW. We focused on absolute changes in measures of MM, rather than comparative changes versus other interventions or placebo, as MM alterations are often associated with changes in weight. As similar weight loss cannot be expected between GLP1RAs and other interventions, a relevant comparison of MM changes cannot be performed. Thus, we opted to assess absolute changes and relate these to changes observed in FM and TBW.

### 2.1 Search strategy and study selection criteria

A comprehensive electronic search was performed by one author (O. A.) on 15th November 2023 for all studies published until that date in the Medline, EMBASE, PubMed, CINAHL, and Cochrane Central Register of Controlled Trials (CENTRAL) databases. Google Scholar was also searched for the identification of gray area literature relevant to the review topic on the same date. The choice of search terms was informed by a prior informal evaluation of the literature performed during the development of the review protocol. The general terms used in the search are listed below and were adapted to each individual database, with mapping to MeSH or EMTREE terms wherever possible and appropriate.

- Population: overweight OR diabet* OR obes*
- Intervention: albiglutide OR lixisenatide OR dulaglutide OR exen* OR semaglutide OR liraglutide OR GLP1RA OR glucagon like peptide 1 receptor agonist
- Outcomes: muscle mass OR fat mass OR fat‐free mass OR lean body mass OR body composition

The Boolean command “AND” was used to combine the sets of terms for the final search. Examples of the search strategy can be found in the Supplementary Material (Appendix S3‐S5).

Studies inclusion criteria were:

1. full article written in English and published in a peer‐reviewed journal
2. population mean body mass index (BMI) ≥ 25 kg.m^‐2^
3. population mean age ≥ 40 years
4. at least one intervention group treated with any GLP1RA for ≥ 6 weeks without any concurrent intervention
5. reporting an outcome related to MM – for example, LBM, FFM, skeletal muscle mass (SMM)

It is important to note that for the primary outcome, any measure that is used to estimate MM met our inclusion criteria. Whilst there are key differences in the exact entities being assessed in these measures,^41^ we also recognized the value in including as broad a range of data sources as possible. Sensitivity analysis was performed, as described subsequently, to determine whether these differing measures introduced substantial heterogeneity to the observed results. For ease, in the following sections of this review, these measures have been incorporated within the broad term of MM.

FM and TBW were secondary outcomes, thus reporting of data related to these outcomes was not essential for inclusion of the study. Both randomized controlled trials (RCT) and non‐RCT studies were included, with the essential requirement that at least one intervention group was treated with a GLP1RA, without any concurrent intervention (e.g. exercise). Studies involving individuals diagnosed with type 1 diabetes, or pregnant, neonatal, pediatric, or adolescent populations were excluded.

We elected to limit the mean age to ≥ 40 years as age‐related declines in MM occur from approximately this age onwards, thus this population is at higher risk from further accelerations in this process. Additionally, below this age MM naturally increases,^32^ thus inclusion of younger cohorts could introduce significant heterogeneity into the review findings.

The full list of citation titles was collated onto a Microsoft Excel (version 2409, Microsoft, Washington, USA) spreadsheet and then manually examined by one author (O. A.) for duplicates that were removed. Following this, two authors (O. A. and S. R.) independently screened the titles and abstracts of the studies identified from the primary search. Disagreements regarding inclusion or rejection at this stage were discussed, with review by a third author (I. I.) in cases where consensus could not be achieved. Inter‐observer agreement rate was 91%, with Cohen's Κ 0.44, suggesting moderate concordance. Articles that addressed the research question but did not meet the specific inclusion criteria were selected as secondary sources. The reference lists of these secondary sources were manually reviewed by one author (O. A.) for the identification of additional potentially relevant studies. Following the completion of screening, full articles from both primary and secondary searches were assessed for eligibility concurrently by two authors (O. A. and S. R.) with disagreements resolved as above.

### 2.2 Data collection and validity assessment

Data collection was performed by a single independent author (O. A.) and assembled into a data extraction spreadsheet (Microsoft Excel version 2409, Microsoft, Washington, USA), which was cross‐checked by another author (S. R.) for accuracy. Pre‐ and post‐intervention measures of the primary outcome (e.g. LBM, FFM, SMM) and the secondary outcomes (FM and TBW, if available) were extracted from each included article. In addition to this, the following information regarding each study was obtained: GLP1RA medication(s) dose(s) and frequency, intervention duration, mean cohort age and BMI, participant gender, diabetes status, study authors, publication date, study design, comparator intervention, and dose (if applicable), and main study outcomes.

In publications where more than one intervention group met the criteria for inclusion in the analysis, each group was recorded independently. Where data were not available in a form that was amenable to meta‐analysis, corresponding authors were contacted requesting additional data. Up to three attempts were made with six weeks between each contact. If no reply was received despite multiple contact attempts, study results were included in the narrative synthesis only.

The method of bias assessment employed was dependent on the study design. For RCTs, the Cochrane Risk of Bias 2 (ROB2) tool was utilized,^42^, ^43^ whilst the relevant Joanna Briggs Institute Critical Appraisal Checklist was implemented for non‐RCTs.^44^ Bias assessments were performed independently by two authors (O. A. and S. R.), and disagreements were resolved by a third author (I. I.). RCTs received a “low”, “some concerns” or “high risk of bias” rating based on five bias domains (randomization process, deviation from intended intervention, missing outcome data, measurement of the outcome, and selection of the reported results). Non‐RCTs received a global rating of quality based on responses to a checklist of questions, which was decided upon the collaboration of the two authors.

### 2.3 Statistical analysis

StataSE version 18 (StataCorp, Texas, USA) was used to perform meta‐analysis of available data. Pre‐ and post‐intervention means and standard deviations (SD) from each study/intervention group were used to generate a weighted mean difference (WMD) for the outcomes. All measures were reported in kilograms, therefore enabling the use of WMD as the effect‐size measure. The random effects Hedges model was selected as we anticipated a potential of significant heterogeneity due to the different medications and doses, and likely variations in population characteristics.

We initially intended to perform subgroup analysis according to diabetes status, due to the expected heterogeneity between these two populations. However, due to the large number of eligible articles identified, a post‐hoc decision was made to perform two separate analyses of studies involving participants with T2DM/prediabetes and studies involving individuals without T2DM. We decided to divide the analysis in this manner as different doses of GLP1RA are utilized for the management of T2DM and obesity without T2DM. Thus, this approach would remove a potentially major source of heterogeneity from the resulting quantitative synthesis.

Forest plots were generated for each analysis and sub‐grouped by GLP1RA. In order to provide context to any changes in measures of MM, forest plots with these measures were presented alongside the corresponding forest plots for FM and TBW. Therefore, two groups of paired forest plots were generated (MM:FM, and MM:TBW), only including data from studies that reported both measures. Test for subgroup was performed when two or more data points were present.

Where studies only reported standard error, SD was calculated in line with Cochrane guidance.^45^ In situations where only a pre‐ or post‐intervention measure and change value have been reported, and the required information was not made available upon request, the missing measure was calculated using the change value, and the SD was assumed to be the same as the available measure. Sensitivity analysis was performed to examine whether this influenced the overall results by analyzing the results obtained when only studies containing both pre‐ and post‐intervention data were included. Sensitivity analysis was also performed to determine the effect of including non‐RCTs.

As there were multiple measures of MM (with important differences in the body compartments being examined), as well as multiple methods used to determine body composition, these were also subject to sensitivity analysis to provide more certainty to the robustness of our results. Finally, to examine whether the inclusion of studies investigating the oral formulation of Semaglutide influenced results, a comparison between oral and subcutaneous Semaglutide was performed.

Study heterogeneity was assessed using I^2^ and Tau statistics (interpreted in accordance with Cochrane guidance), as well as visual examination of the generated forest plots. Evidence of homogeneity was assessed using the H^2^ and test for Θ_i_. Evidence of publication bias was examined through visual inspection of funnel plots and the performance of the Egger test. Statistical significance was defined as p < 0.05 and data are presented as WMD with 95% confidence intervals.

## 3 RESULTS

### 3.1 Literature search and study characteristics

**FIGURE 1:**
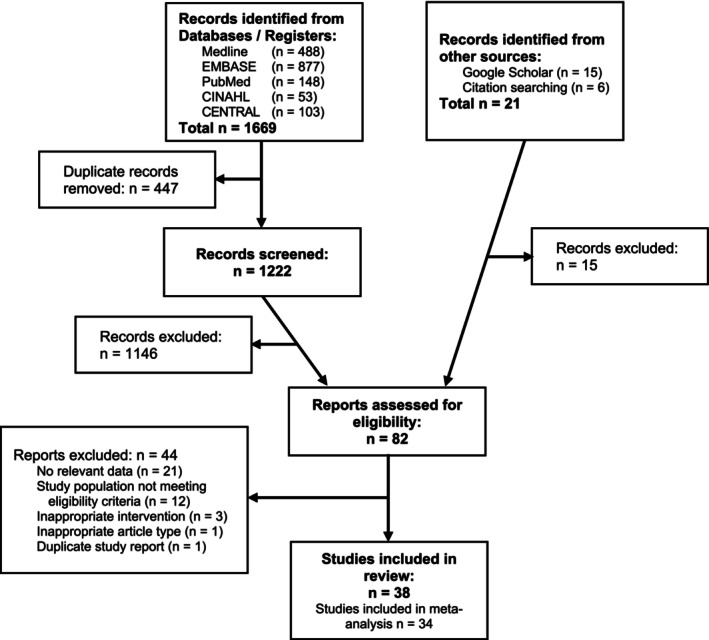
Flow diagram of study selection.

An overview of the entire study selection process is outlined in Figure 1. Initial search of the five databases identified 1222 unique citations, following the removal of duplicates. Google Scholar search yielded 15 relevant citations, and manual reviewing of secondary sources provided a further six citations. The full texts of 82 publications were reviewed, of which 44 were excluded at this stage. Thus, 38 studies were included in this systematic review and their characteristics are presented in Table 1. Four studies did not contain data amenable to meta‐analysis, due to reporting indices of FM and FFM,^67^ FFM and FM percentages,^47^ or interquartile ranges.^63^, ^81^ Therefore 34 were included in the quantitative synthesis.

**TABLE 1:** Characteristics of studies included in the review.

Study	Study design	GLP1RA & dose	Duration	Sample size & gender (no. of females)	Diabetes status	Age – mean (SD)	Baseline BMI – mean (SD)	Method of body composition assessment	Outcomes of interest measured	Study primary outcome/s
**van Eyk et al, 2019** ^46^	RCT	Liraglutide, 1.8 mg od	26 weeks	22 (14)	T2DM	55 (11)	30.4 (3.8)	BIA	LBM, TBW	Cardiac function
**Diaz‐Soto et al., 2014** ^47^	Non‐RCT (SG)	Liraglutide, 1.8 mg od	14 weeks	55 (27)	T2DM	61 (11)	33.9 (5.7)	BIA	FFM% /FM% /BMI	Undisclosed
**Yabe et al., 2022** ^48^	RCT	Dulaglutide, 0.75 mg ow	52 weeks	19 (3)	T2DM	58.6 (7.5)	27.5 (3.5)	BIA	LBM /FM/TBW	Medication pharmacodynamics
**Li et al., 2014** ^49^	Non‐RCT (SG)	Liraglutide, 1.2 mg od	12 weeks	31 (15)	T2DM	48.5 (11.4)	31.7 (3.6)	DXA	LBM /FM/TBW	Change in cardiac natiuretic peptides
**Stefanakis et al., 2023** ^50^	Non‐RCT (MG)	Liraglutide, 3 mg od	24 weeks	9 (3)	Non‐DM	44.6 (7.4)	37.9 (4.6)	BIA	FFM /FM/TBW	Postprandial C‐peptide & proglucagon‐derived peptides
**Park et al., 2021** ^51^	Non‐RCT (SG)	Liraglutide, 3 mg od	Variable, 74 days average	169 (98)	Mixed	41.5 (9.6)	30.8 (3.5)	BIA	SMM /FM/TBW	Undisclosed
**Agcakaya et al., 2023** ^52^	Non‐RCT (SG, retrospective)	Exenatide, 10mcg bd	6 months	50 (46)	T2DM	56.3 (9.7)	35.3 (7.6)	BIA	FFM% /FM% /TBW	Weight loss & body composition
**Seko et al., 2017** ^53^	Non‐RCT (SG, retrospective)	Dulaglutide, 0.75 mg ow	12 weeks	5 (undisclosed)	T2DM	66.8 (2.7)	28.2 (1.2)	BIA	SMM /FM/TBW	Change in liver function & fibrosis markers
**Freitas et al., 2023a** ^54^	Cohort study	Liraglutide, 3 mg od	6 months	24 (all female)	Mixed	46 (9)	36.3 (3.9)	BIA	SMM /FM/TBW	Undisclosed
**Freitas et al., 2023b** ^54^	Cohort study	Liraglutide, 3 mg od	6 months	33 (all male)	Mixed	42 (11)	38.3 (5.8)	BIA	SMM /FM/TBW	Undisclosed
**Silver et al., 2023** ^55^	RCT	Liraglutide, 1.8 mg od	14 weeks	44 (31)	Pre‐DM	49.8 (10.1)	38.6 (6.1)	DXA	LBM /FM/TBW	Appetite, food intake & fat distribution
**Yin et al., 2018** ^56^	RCT	Exenatide, 10mcg bd	16 weeks	19 (7)	T2DM	47.6 (10.9)	28.1 (2.2)	DXA	LBM /FM/TBW	HbA1c
**Neeland et al., 2021** ^57^	RCT	Liraglutide, 3 mg od	40 weeks	73 (67)	Non‐DM	49.6 (9.8)	37.2 (6.0)	MRI based measure	Lean tissue /adipose tissue/TBW	Visceral adipose tissue
**Feng et al., 2019** ^58^	RCT	Liraglutide, 1.8 mg od	24 weeks	29 (8)	T2DM	46.8 (9.7)	28.1 (3.2)	DXA	LBM /FM/TBW	Weight
**Blundell et al., 2017** ^12^	Crossover RCT	Semaglutide, 1 mg ow	12 weeks	28 (18)	Non‐DM	42 (range 21–70)	33.8 (range 30.5–42.8)	ADP	LBM /FM/TBW	Change in energy intake
**Gibbons et al., 2021** ^59^	Crossover RCT	Semaglutide, 14 mg od	12 weeks	13 (2)	T2DM	58.2 (no SD)	30.8 (no SD)	ADP	LBM /FM/TBW	Postprandial glucose metabolism
**Hong et al., 2016** ^60^	Non‐RCT (SG)	Exenatide, 10mcg bd	12 weeks	32 (undisclosed)	T2DM	49.0 (11.2)	32.9 (4.7)	BIA	SMM /FM/TBW	Undisclosed
**de Luis et al., 2015a** ^61^	Non‐RCT (MG)	Liraglutide, 1.8 mg od	14 weeks	51 (26)	T2DM	60.5 (9.2)	34.9 (6.6)	BIA	FFM /FM/TBW	Body weight
**de Luis et al., 2015b** ^61^	Non‐RCT (MG)	Liraglutide, 1.8 mg od	14 weeks	39 (18)	T2DM	61.2 (10.1)	33.1 (4.9)	BIA	FFM /FM/TBW	Body weight
**Bunck et al., 2010** ^62^	RCT	Exenatide, 20mcg tds	52 weeks	29 (8)	T2DM	58.4 (7.5)	30.9 (3.8)	DXA	LBM /FM/TBW	Beta‐cell function
**Kadouh et al., 2020** ^63^	RCT	Liraglutide, 3 mg od	16 weeks	17 (16)	Non‐DM	42 (IQR 32–51)	37.2 (IQR 33.6–41.0)	DXA	LBM/FM% /TBW	Undisclosed
**McCrimmon et al., 2020** ^64^	RCT	Semaglutide, 1 mg ow	52 weeks	88 (undisclosed)	T2DM	57.8 (9.9)	32.6 (6.4)	DXA	LBM /FM/TBW	Body composition
**Lundgren et al., 2021** ^65^	RCT	Liraglutide, 1 mg od	52 weeks	41 (23)	Non‐DM	43 (12)	32.7 (3.1)	DXA	LBM /FM/TBW	Body weight
**Perna et al., 2016** ^66^	Non‐RCT (SG)	Liraglutide, 3 mg od	24 weeks	9 (3)	T2DM	68.2 (3.9)	32.4 (4.9)	DXA	FFM /FM/TBW	Body composition
**Ishii et al., 2019** ^67^	Non‐RCT (SG)	Liraglutide, 0.9 mg od	24 weeks	9 (5)	T2DM	44.7 (12.2)	37.4 (6.4)	DXA	SMI /FI/BMI	Body composition
**Santini et al., 2023** ^68^	Non‐RCT (SG)	Liraglutide, 3 mg od	10 months	36 (7)	Non‐DM	43.6 (11.6)	40.8 (5.7)	DXA	LBM /FM/TBW	Body weight
**Wilding et al, 2021** ^20^	RCT	Semaglutide, 2.4 mg ow	68 weeks	95 (72)	Non‐DM	50 (12)	34.8 (3.6)	DXA	LBM/FM	Percentage weight change
**Volpe et al., 2022 – 1** ^69^	Non‐RCT (SG)	Semaglutide, 0.5 mg ow	26 weeks	40 (19)	T2DM	64.9 (10.8)	38.8 (7.7)	BIA	SMM/FMI/TBW	Body composition & weight
**Volpe et al., 2022 – 2** ^70^	Non‐RCT (SG)	Semaglutide, 1 mg ow	52 weeks	48 (22)	T2DM	57.7 (8.4)	38.8 (8.3)	BIA	SMI /FMI/TBW	Liver function and appearance
**Volpe et al., 2023** ^71^	Non‐RCT (SG)	Semaglutide, 7 mg od	26 weeks	32 (18)	T2DM	66.3 (8.5)	28.2 (3.3)	BIA	FFM /FM/TBW	Body composition
**Uchiyama et al., 2023** ^72^	Non‐RCT (SG)	Semaglutide, 14 mg od	24 weeks	25 (11)	T2DM	54.1 (13.5)	29.3 (3.4)	BIA	LBM /FM/BMI	Body composition
**Iepsen et al., 2018** ^73^	Non‐RCT (MG)	Liraglutide, 3 mg od	16 weeks	28 (12)	Non‐DM	42.8 (10.2)	36.8 (4.8)	DXA	LBM /FM/TBW	Body weight
**de Luis et al., 2014a** ^74^	Non‐RCT (MG)	Liraglutide, 1.8 mg od	14 weeks	51 (26)	T2DM	48.9 (16.3)	34.1 (6.9)	BIA	FFM /FM/TBW	Body weight
**de Luis et al., 2014b** ^74^	Non‐RCT (MG)	Liraglutide, 1.8 mg od	14 weeks	35 (16)	T2DM	47.3 (16.4)	31.8 (5.1)	BIA	FFM /FM/TBW	Body weight
**Astrup et al., 2012a** ^75^	RCT	Liraglutide, 1.2 mg od	20 weeks	15 (undisclosed)	Non‐DM	47.2 (9.7)	34.8 (2.6)	DXA	LBM/FM	Weight change
**Astrup et al., 2012b** ^75^	RCT	Liraglutide, 1.8 mg od	20 weeks	13 (undisclosed)	Non‐DM	45.5 (10.9)	35.0 (2.6)	DXA	LBM/FM	Weight change
**Astrup et al., 2012c** ^75^	RCT	Liraglutide, 2.4 mg od	20 weeks	15 (undisclosed)	Non‐DM	45.0 (11.1)	35.0 (2.6)	DXA	LBM/FM	Weight change
**Astrup et al., 2012d** ^75^	RCT	Liraglutide, 3.0 mg od	20 weeks	15 (undisclosed)	Non‐DM	45.9 (10.7)	34.8 (2.8)	DXA	LBM/FM	Weight change
**Harder et al., 2004** ^76^	RCT	Liraglutide, 0.6 mg od	8 weeks	21 (10)	T2DM	59.9 (11.0)	36.8 (4.6)	DXA	LBM /FM/TBW	Undisclosed
**Ozeki et al., 2022** ^77^	Non‐RCT (SG, retrospective)	Semaglutide – dose undisclosed	3 months	13 (undisclosed)	T2DM	52.0 (6.9)	35.9 (6.1)	BIA	SMM /FM/TBW	Body composition
**Heise et al., 2023** ^78^	RCT	Semaglutide, 1 mg ow	28 weeks	44 (10)	T2DM	63.7 (5.9)	30.8 (3.8)	ADP	FFM /FM/TBW	Beta‐cell function & insulin sensitivity
**Rondanelli et al., 2016** ^79^	Non‐RCT (SG)	Liraglutide, 3 mg od	6 months	28 (12)	T2DM	58.8 (9.3)	34.1 (5.5)	DXA	FFM /FM/TBW	Body composition
**Jendle et al., 2009a** ^80^	RCT	Liraglutide, 1.2 mg od	52 weeks	23 (11)	T2DM	55 (11)	Undisclosed	DXA	LBM /FM/TBW	Body composition
**Jendle et al., 2009b** ^80^	RCT	Liraglutide, 1.8 mg od	52 weeks	20 (10)	T2DM	54 (9)	Undisclosed	DXA	LBM /FM/TBW	Body composition
**Timofte et al., 2015a** ^81^	Non‐RCT (MG)	Exenatide – dose undisclosed	6 months	112 (45)	T2DM	58.7 (11.4) /58.1 (12.1)	37.3 (IQR 33.2–42.4)	BIA	FFM /FM/TBW	Undisclosed
**Timofte et al., 2015b** ^81^	Non‐RCT (MG)	Liraglutide – dose undisclosed	6 months	98 (36)	T2DM	58.5 (12.6) /57.5 (11.4)	36.8 (IQR 33.1–41.2)	BIA	FFM /FM/TBW	Undisclosed

RCT – randomized controlled trial, Non‐DM – without diabetes, Pre‐DM – prediabetic, SG – single group, MG – multiple group, IQR – interquartile range, BIA – bioimpedance analysis, DXA – dual‐energy x‐ray absorptiometry, MRI – magnetic resonance imaging, ADP – air displacement plethysmography, SMM – skeletal muscle mass, od – once daily, bd – twice daily, tds – three times daily, ow – once weekly.

Of the 38 studies included, 17 were RCTs (including two crossover RCTs), seven were non‐randomized multiple‐group studies, and 14 were single‐cohort studies. The 38 studies included a total of 1735 participants, although the number in each study ranged from five to 169. Thirty‐five of the studies reported mixed‐gender cohorts and one study segregated males and females into separate groups. In two studies the gender of participants was not disclosed. Six studies contained more than one intervention group that fulfilled the inclusion criteria, and the data were presented as separate cohorts. Five of the six studies contained two eligible cohorts, and one study contained four. In total, 46 eligible cohorts were included in the review, 41 of which were incorporated into the quantitative synthesis.

Twenty‐seven studies included individuals with T2DM or pre‐DM, and 24 were included in the meta‐analysis. Eleven studies containing 15 cohorts were included in the review of studies involving participants without T2DM, with 10 studies (14 cohorts) included in the “non‐DM” meta‐analysis. Of these studies, two had populations that included a mixture of individuals with and without T2DM. However, in both cases, the majority of participants were not diagnosed with T2DM, the mean baseline HbA1c was in the normal range, and the dose of GLP1RA used is more commonly associated with non‐DM weight management. Thus, both studies were included in the non‐DM analysis.

In the T2DM analysis, four studies investigated Exenatide, two studies investigated Dulaglutide, twelve investigated Liraglutide, and eight investigated Semaglutide. One study investigated both Liraglutide and Exenatide in two distinct intervention groups. In the non‐DM analysis, only Liraglutide (nine studies) and Semaglutide (two studies) were investigated. Of the studies included in the meta‐analysis, 33 reported a measure of MM alongside FM (23 T2DM, 10 non‐DM), while 29 reported a measure of MM with TBW (22 T2DM, 7 non‐DM).

### 3.2 GLP1RA effect on measures of MM in individuals with T2DM

**FIGURE 2:**
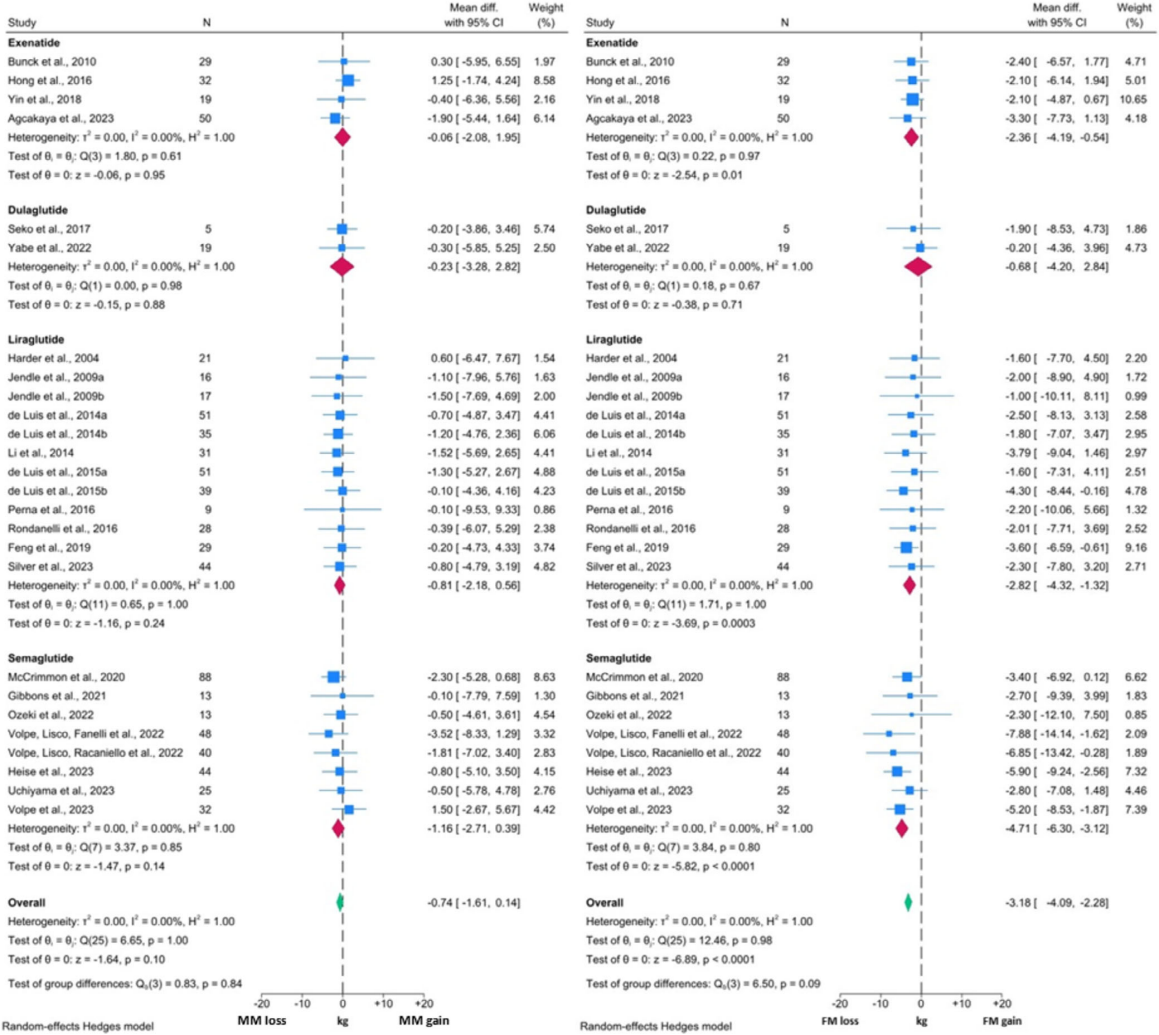
Forest plots showing meta‐analysis results of studies reporting both MM measures and FM in individuals with T2DM.

The 24 studies included in the T2DM meta‐analysis contained a total of 27 cohorts and 850 participants. Figure 2 presents the comparisons between mean change from baseline in MM and FM for each included cohort, sub‐grouped by administered GLP1RA. There was a significant reduction in FM with a WMD of ‐3.18 kg (95% CI: ‐4.09, ‐2.28; p < 0.0001). In comparison, the WMD for MM was ‐0.74 kg (95% CI: ‐1.61, 0.14; p = 0.10). Thus, despite a significant reduction in FM, a non‐significant reduction in MM was observed. This lack of significant change in MM was consistent across the four GLP1RAs that were included in the subgroup analysis. Three out of the four GLP1RAs (Exenatide, Liraglutide, and Semaglutide) elicited significant loss of FM, whilst no significant reduction in FM was observed with Dulaglutide.

**FIGURE 3:**
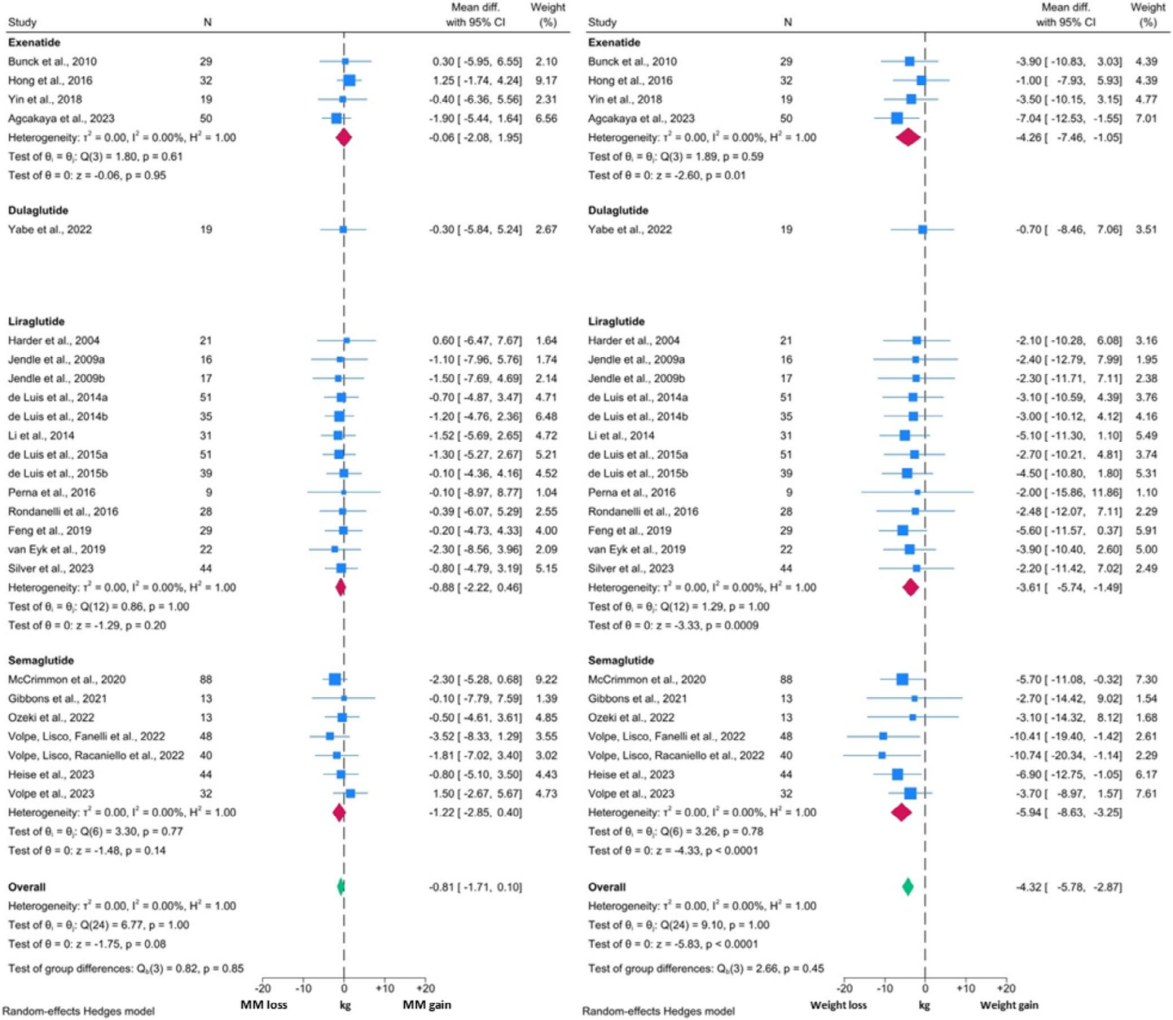
Forest plots showing meta‐analysis results of studies reporting both MM measures and TBW in individuals with T2DM.

The comparison between changes in MM and TBW revealed similar findings (Figure 3). The change in MM from baseline was ‐0.81 kg (95% CI: ‐1.71, 0.10; p = 0.08) which again was not significant. TBW reduced significantly by 4.32 kg (95% CI: ‐5.78, ‐2.87; p < 0.0001). Thus, MM constituted approximately 18.8% of total weight loss in these studies. The reduction in TBW was significant with Exenatide, Liraglutide, and Semaglutide. Only one study investigating Dulaglutide was included in this analysis and the TBW changes were non‐significant.

I^2^ values across all analyses within these two comparisons were 0.00% suggesting the presence of minimal statistical heterogeneity. However, visual inspection of the forest plots identified likely clinical heterogeneity, as represented by a relative excess in the 95% CI range. Further, homogeneity assessments were equivocal for all subgroups (p > 0.05). In both MM forest plots, some studies reported numerical increases in MM, with others reporting reductions. Additionally, whilst all studies reported numerical decreases in FM and TBW, there were variations in the degree of observed reduction. In both cases, this appears to be primarily related to the methodological characteristics of each individual study. For example, the two studies published in 2022 by Volpe *et al* ^69^, ^70^ which reported the greatest reduction in FM and TBW, used Semaglutide as the intervention GLP1RA. On the other hand, Yabe *et al* ^48^ investigated Dulaglutide, which is known to be less effective for weight loss.^82^, ^83^ Additionally, the duration of the intervention may have introduced further heterogeneity. The intervention duration in the study by Harder *et al* ^76^ was eight weeks, whilst Feng *et al* ^58^ administered Liraglutide for 24 weeks, resulting in greater weight loss. Notably, the association between study duration and TBW/FM changes was not always consistent, as the intervention length in the study by Jendle *et al* ^80^ was 52 weeks, which reported similar changes to Harder *et al*.^76^

Three studies could not be included in the meta‐analysis, due to a lack of amenable data. Kadouh *et al* reported a non‐significant 1.3 kg reduction in LBM in the context of a significant 5.8 kg weight loss and a 2 percentage‐point reduction in FM percentage.^63^ Timofte *et al* outlined the effects of both Exenatide and Liraglutide on the body composition of their cohorts.^81^ In contrast to Kadouh *et al*, this study reported significant reductions in FFM, along with decreased FM and BMI, in both GLP1RA groups.^81^ This study observed a numerically greater loss of FM than FFM, however, there was no comment on the significance of this difference.^81^ Finally, Diaz‐Soto *et al* reported changes in FM and FFM percentage, with a significant decrease in FM percentage and a corresponding increase in FFM percentage observed following 14 weeks of Liraglutide treatment.^47^

Taken together, these findings suggest that GLP1RAs, when used in the context of managing T2DM, induce significant reductions in body weight and FM and a non‐significant reduction in measures of MM. Changes in MM measures constituted under 20% of the total weight reduction, resulting in a consequent increase in MM percentage post‐intervention.

### 3.3 GLP1RA effect on measures of MM in individuals without T2DM

**FIGURE 4:**
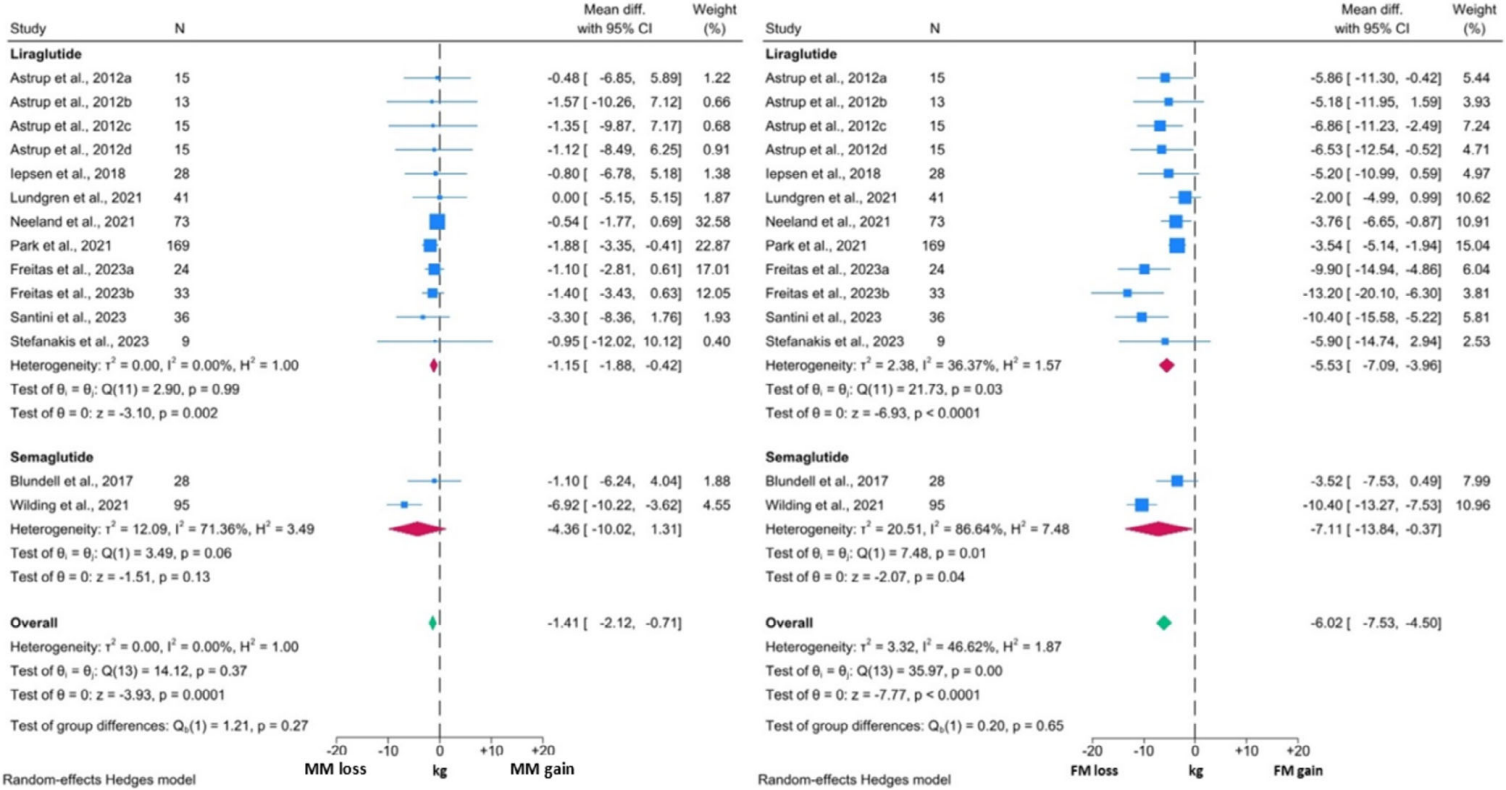
Forest plots showing meta‐analysis results of studies reporting both MM measures and FM in individuals without T2DM.

Ten studies were included in the non‐DM meta‐analysis, which contained 14 intervention cohorts, and 594 participants. The forest plots for the comparison between MM and FM are presented in Figure 4. Quantitative analysis revealed a significant reduction in both MM and FM among these studies. FM reduced by 6.02 kg (95% CI: ‐7.53, ‐4.50; p < 0.0001), whilst MM reduced by ‐1.41 kg (95% CI: ‐2.12, ‐0.71; p = 0.0001). Therefore, the reduction of FM was significantly greater than the MM reduction observed in these studies. Only two GLP1RAs were evaluated in this analysis, and the loss of FM was significant across both. Only Liraglutide demonstrated a significant reduction in MM, whilst a non‐significant reduction was observed with Semaglutide. Notably, only two studies investigated body composition changes with Semaglutide, with substantial heterogeneity between the reported effects.

This heterogeneity is likely due to methodological differences between the studies with Blundell *et al* administering Semaglutide 1 mg for 12 weeks^12^ and Wilding *et al* investigating Semaglutide 2.4 mg for 68 weeks.^20^ This was supported by the elevated I^2^ values in MM and FM analysis for Semaglutide. Furthermore, FM analysis in the Liraglutide subgroup exhibited moderate to substantial heterogeneity, with evidence of limited homogeneity also observed (p < 0.05). The widest variation in FM change was between Freitas *et al* ^54^ and the studies by Neeland *et al* and Lundgren *et al*.^57^, ^65^ This was despite very similar methodologies using Liraglutide 3 mg for 6 – 12 months. Thus, differences in the clinical features of the populations in these studies are likely to account for the heterogeneity noted in this analysis. Interestingly, this heterogeneity was not reflected in the MM analysis, suggesting consistency among these changes, despite large differences in FM alterations.

**FIGURE 5:**
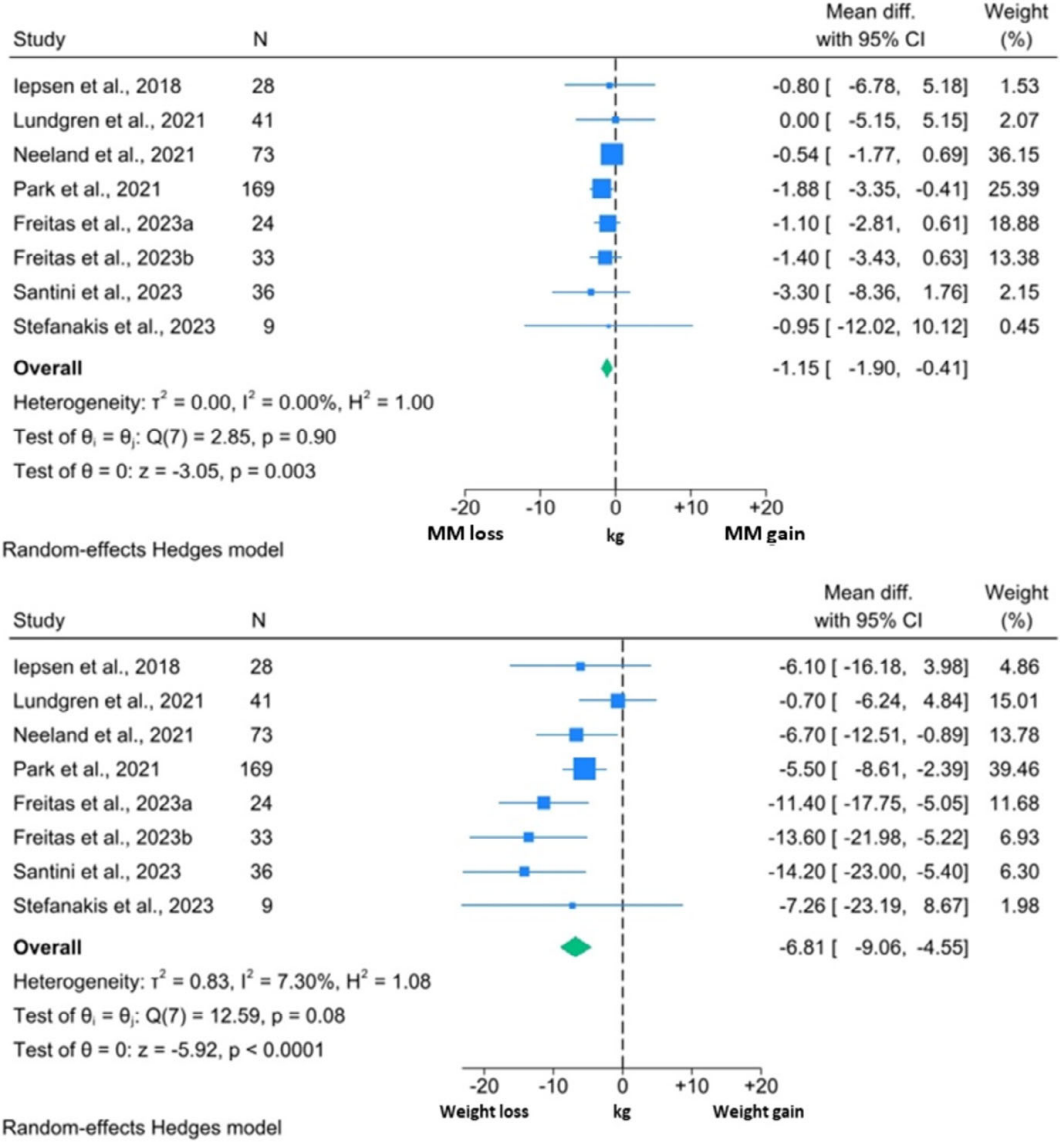
Forest plots showing meta‐analysis results of studies reporting both MM measures and TBW in non‐DM individuals, Liraglutide was the only GLP1RA assessed in this analysis.

Comparisons between changes in MM and TBW were only possible in studies investigating the GLP1RA Liraglutide and these are outlined in Figure 5. Again, both TBW and MM reduced significantly, with Liraglutide inducing a 6.81 kg weight reduction (95% CI: ‐9.06, ‐4.55; p < 0.0001) and a corresponding 1.15 kg reduction in measures of MM (95% CI: ‐1.90, ‐0.41; p = 0.003). Thus, MM comprised of 16.9% of total weight loss experienced by individuals without T2DM. The I^2^ for the MM forest plot was 0.00%, whilst the corresponding value for TBW was 7.30% suggesting heterogeneity might not be important. Visual inspection of the forest plot showed variation in weight reduction, which again is likely reflective of clinical differences between the study populations.

One study by Ishii *et al* was unable to be included in the meta‐analysis.^67^ This study administered Liraglutide 0.9 mg once daily for 24 weeks, and calculated indices of SMM and FM relative to height.^67^ The authors reported a significant reduction in fat index and BMI however, this was associated with a non‐significant reduction in skeletal muscle index.^67^

Overall, contrary to the results observed in the T2DM analysis, GLP1RAs induced significant reductions in MM in individuals without T2DM. However, this again was significantly less than the reduction in FM. Comparison between changes in MM and TBW was limited to studies investigating Liraglutide, however similar to the T2DM analysis, MM comprised a small proportion of total weight loss in the included studies.

### 3.4 Sensitivity analysis

To assess the impact of including studies that did not report both pre‐intervention and post‐intervention measures on the results, we performed a sensitivity analysis, including only studies that contained a full data set. The results are presented in Table S1, and were consistent with those from the main analysis, suggesting that inclusion of these studies did not have a significant effect on the observed results.

Secondly, we examined the impact of including non‐RCTs by performing an analysis including only RCT studies (Table S2). For the T2DM studies, the results were consistent with the main analysis. For non‐DM studies, the findings of the MM:FM comparison were again consistent with the main analysis. This was not the case with the MM:TBW comparison in which, contrary to the main analysis findings, there was no significant change in either parameter. Notably, only two studies were eligible for inclusion in this sensitivity analysis, and their results were markedly heterogenous. The study by Lungren *et al* ^65^ in particular reported minimal changes in LBM and weight. This non‐significant change in weight is inconsistent with established literature on GLP1RAs in individuals without T2DM, thus the skewing of this sensitivity analysis by this study likely accounts for the observed discrepancy.

We also performed a sensitivity analysis to determine whether the method of measuring body composition and the measure of MM reported by studies may have influenced the results. Regardless of the three methods of body composition analysis chosen, the changes in MM and FM were consistent with those of the main findings (Table S3). Similarly, whether FFM, LBM, or SMM were reported as measures of MM, the findings were similar in T2DM and non‐DM studies (Table S4). Thus, these two factors did not significantly influence the results obtained.

Finally, sensitivity analysis investigating potential differences between oral and subcutaneous Semaglutide is reported in Table S5. Oral Semaglutide was only utilized in studies included in the T2DM analysis, and not all of these studies reported weight outcomes. There were no significant differences identified in the changes in measures of MM or FM (test for group differences p = 0.18 and p = 0.51, respectively).

### 3.5 Risk of bias assessments

Quality assessments for RCTs were performed using the Cochrane ROB2 tool and are displayed in Figures S1 & S2. Five of the 15 standard RCT studies were rated as “low” ROB. The remaining 10 studies were classified as having “some concerns”. This is primarily due to concerns noted in domain 5, arising from a lack of information expressing a pre‐specified plan for analyzing body composition outcomes. Two studies did not explicitly state whether body composition assessors were blinded to the allocation of participants and therefore were rated “some concerns” in domain 4. Finally, one study had a concern related to domain 1 caused by inability to confirm if the allocation sequence was concealed.

The two crossover RCTs were assessed with the ROB2 tool specific to this type of study. Both studies were rated with “some concerns” in domains 1 and 5, arising from a lack of information regarding the performance and concealment of the randomized allocation sequence, and uncertainty about a pre‐specified plan for outcome analysis.

The quality of the 14 non‐RCT studies was assessed using checklists developed by the Joanna Briggs Institute, which enable an overall judgment of study quality and are presented in Tables S6 & S7. Eight of the studies exhibited good quality. The remaining studies exhibited methodological constraints reducing their quality status. Considering the global assessment of each study, one^60^ received “no” or “unclear” answers to five out of 10 questions, and therefore was identified as reduced study quality based on this model of assessment.^64^

Of the seven multiple‐group non‐RCT studies, four received a “yes” answer to all relevant questions, suggesting good quality. The remaining three produced “no” answers to at least one question, raising concerns regarding their quality. Nonetheless, all studies received “yes” answers to the majority of questions, and therefore the overall quality of the included studies was considered acceptable.

### 3.6 Publication bias assessment

Funnel plots and the Egger test were performed for T2DM and non‐DM studies separately. Both funnel plots demonstrated good symmetry around the mean value implying a lack of publication bias (Figures S3 & S4). In agreement with this, the Egger test was non‐significant (T2DM p = 0.95, non‐DM p = 0.48), providing further evidence of a lack of publication bias within our analysis.

## 4 DISCUSSION

This systematic review and meta‐analysis has demonstrated novel findings with regard to the impact of GLP1RAs on measures of MM, dependent on glycaemic status. In individuals with T2DM, GLP1RAs induce a non‐significant reduction in MM measures, despite corresponding significant reductions in TBW and FM. In individuals without T2DM, a significant reduction in MM measures was observed, however, this was to a significantly lower extent than the associated FM reduction in these individuals. Furthermore, our statistical analysis of both populations revealed that MM comprised less than 20% of the total observed weight loss in the included studies.

T2DM is associated with accelerated age‐related loss of muscle mass,^33^, ^84^ whilst the cycles of weight loss and weight regain among individuals living with obesity predispose to sarcopenic obesity.^85^ Both of these pathological states significantly increase the risk of disability and death.^86^, ^87^ Weight loss interventions are a critical aspect of managing T2DM and obesity, however, concerns exist regarding the associated loss of MM.^35^ Whilst measures such as adjuvant resistance training or protein supplementation have emerged as potential methods of ameliorating MM reduction in this context, interventions that induce weight loss while preserving MM remain highly sought after.^23^

Several authors have hinted at the potential ability of GLP1RAs to preserve MM during weight loss in individuals with T2DM.^37^, ^88^, ^89^ The authors of a narrative review on this topic concluded that GLP1RAs and SGLT2‐inhibitors (another group of diabetes medications) were associated with reductions in LBM.^36^ However, they also highlight that as LBM losses were of a lower magnitude to those observed in FM, participants had a more favorable body composition post‐intervention.^36^ A network meta‐analysis of multiple agents used for T2DM management suggested that GLP1RAs, particularly Semaglutide, were associated with loss of FFM, and stressed the importance of considering this when prescribing these medications.^22^

In agreement with the suggestions of Zhang *et al*,^37^ our findings add objective systematic evidence to the proposition that GLP1RAs may act to preserve MM in individuals with T2DM in the context of weight loss. The present systematic review and meta‐analysis is the only one to assess this quantitatively, across a range of GLP1RAs and various study types. We felt this was essential to ensure the broadest range of information was considered. Our sensitivity analysis revealed that similar findings would have been derived if only RCTs were included, adding further strength to our conclusions.

In non‐DM studies, while the reduction in MM measures was significant, the mean 1.15 kg loss was a small proportion of the total 6.81 kg weight loss observed across those studies. This contrasts with bariatric surgery, where a meta‐analysis has reported a 9.74 kg reduction in FFM in the context of 25.65 kg FM loss.^90^ This equates to almost 30% of total weight loss, although it is important to note that the studies in the analyses performed were not matched. Meanwhile, a systematic review of FFM losses with caloric restriction interventions estimated that 25% of total weight lost was derived from FFM, although this was not quantitively assessed, and total weight loss from these interventions was not reported.^91^ In a study performed by our group, LBM accounted for almost 40% of total weight lost following a very‐low‐calorie diet intervention in overweight individuals without T2DM.^92^

Thus, our findings suggest a lower MM proportion of total weight loss with respect to GLP1RAs, compared to bariatric surgery and dietary interventions. This observation implies a possible muscle‐preserving effect of GLP1RAs, although it is important to note that a direct comparison between GLP1RAs and other modalities of weight loss intervention has not been performed. Furthermore, a detailed exploration of the mechanisms underlying this potential effect is not within the scope of this review. Nevertheless, this assertion is supported by the widespread expression of GLP‐1 hormone receptors in skeletal muscle cells.^93^ Moreover, we recently reported that myofibrillar muscle protein synthesis (MPS) under postprandial conditions increased in the presence of GLP‐1 hormone infusion, along with a consequent rise in plasma insulin concentrations.^39^

It could be speculated that this effect is mediated by insulin, given that GLP‐1 stimulates insulin secretion,^94^ and insulin has been associated with increased MPS.^95^, ^96^ However, our previous meta‐analysis concluded that insulin does not significantly increase MPS, but does suppress muscle protein breakdown (MPB), with an associated increase in net protein balance.^97^ Thus, GLP‐1 hormone could modulate MPB via insulin‐dependent mechanisms, rather than MPS. Furthermore, in a rodent study, the GLP1RA exendin‐4 was found to ameliorate muscle wasting and recover muscle mass through direct suppression of atrophic factors and enhancement of myogenic factors.^40^ Taken together, GLP1RAs appear to inhibit MPB directly or indirectly via insulin and appear to also stimulate MPS via insulin‐independent pathways, consequently leading to an overall protective effect on MM.

The significant loss of FM along with weight loss was not surprising and has been observed in previous reviews.^36^ A meta‐analysis quantitatively demonstrated significant reductions in both visceral and subcutaneous adipose tissue when compared to placebo in individuals with T2DM.^98^ Contrastingly, another meta‐analysis failed to demonstrate significant reductions in FM with Liraglutide.^99^ However, only two studies were included in the quantitative analysis, which did not demonstrate significant weight loss, limiting the conclusions that could be drawn.^99^ The weight loss reported by the present review was comparable to that observed with GLP1RAs in the context of T2DM^100^, ^101^, ^102^ and obesity.^103^

Our meta‐analysis was limited by the lack of placebo‐controlled RCTs exploring this issue, necessitating the inclusion of data from non‐RCT studies and the calculation of pre‐post intervention changes. Our sensitivity analysis suggested that the inclusion of non‐RCT did not significantly alter the obtained results. As body composition analysis is usually automated, it is unlikely that lack of randomization or blinding would have introduced significant methodological bias to the findings.

One important and highly relevant limitation of this review is that multiple measures of MM estimation were included. Whilst LBM, FFM, SMM, and lean tissue are widely utilized as surrogate markers of MM, they have important differences in how they are determined.^104^ These differences are likely to result in varying degrees of inaccuracy when estimating true changes in functional skeletal muscle mass. Sensitivity analysis demonstrated similar findings regardless of the measure reported, providing some reassurance to the robustness of the results, however, these potential differences cannot go unrecognized.

In relation to this, the vast majority of included studies used non‐gold standard techniques (DXA, BIA, or ADP) for estimating MM. These methods are prone to over‐estimating changes in MM as they detect fat‐free/lean tissue in all body compartments, including adipose tissue.^41^ Thus, large reductions in adipose tissue will result in reductions in FFM or LBM, even if there is absolutely no loss of skeletal muscle.^41^ It is therefore likely that the true reductions in functional skeletal muscle are less than what has been reported in this review, as was observed by Bosy‐Westphal *et al*.^105^ Only one study used MRI techniques, whilst none used established methods of directly determining MM, such as D3‐creatine. Again, sensitivity analysis did not reveal any differences in the observed results between the various methods, however, it is important to note both of these limitations when interpreting the results of this review.

Mild to moderate heterogeneity was detected within some of our analyses, which has likely arisen from differences in intervention doses, intervention durations, and clinical characteristics (such as age, gender, and baseline BMI) of participants in the included studies. The potential effects of these variables were not assessed and could have added further strength to our conclusions. Finally, this review did not include the exploration of potential mechanisms underlying GLP1RA‐induced preservation of muscle mass. Therefore, further placebo‐controlled studies assessing one consistent measure of MM derived from gold‐standard methodologies, along with an assessment of the potential effect of various population and intervention characteristics would enable the performance of a more rigorous review, with further studies examining mechanisms of GLP1RA action on muscle metabolism providing greater qualification of the observed findings.

In conclusion, this meta‐analysis demonstrated that when used in individuals with T2DM, GLP1RAs do not induce a significant reduction in measures used to estimate muscle mass, despite significant reductions observed in weight and fat mass. In individuals with obesity, a significant reduction in muscle mass measures was observed, however, this was significantly less than the corresponding reduction in fat mass. In both cases, the mean reduction in measures of muscle mass constituted less than 20% of total weight loss, which appears to be less than the amount reported from bariatric surgery and dietary interventions. The reduction in weight in these studies was comparable to that observed in other studies of GLP1RAs, suggesting our findings are robust. However, the widespread use of non‐gold standard techniques for determining skeletal muscle mass in the included studies may have resulted in an overestimation of the true changes in functional muscle mass. Overall, this review has provided useful information that is pertinent to clinicians involved in the management of T2DM and obesity, however, further studies are required to qualify the reported findings.

## CONFLICT OF INTEREST STATEMENT

The authors have no conflicts of interest to disclose in relation to this work.

## Supporting information

Appendix S1: PRISMA Checklist
Appendix S2: Protocol for Systematic Review/Meta‐Analysis
Appendix S3: Medline search strategy
Appendix S4: Pubmed search strategy
Appendix S5: EMBASE search strategy
Table S1: Sensitivity analysis – results from studies reporting full data set only
Table S2: Sensitivity analysis – results from RCT studies only
Table S3: Sensitivity analysis – result by method of body composition assessment
Table S4: Sensitivity analysis – result by measure of SM mass
Table S5: Sensitivity analysis – subcutaneous v oral Semaglutide
Figure S1: Risk of bias assessment results for RCT studies
Figure S2: Risk of bias assessment results for Crossover RCT studies
Table S6: Risk of bias assessments for single group non‐RCT studies.
Table S7: Risk of bias assessment for multiple group non‐RCT studies
Figure S3: Funnel plot of T2DM studies reporting measures of MM
Figure S4: Funnel plot of non‐DM studies reporting measures of MM
