# Supplementary material for: How do glucagon‐like Peptide‐1 receptor agonists affect measures of muscle mass in individuals with, and without, type 2 diabetes: A systematic review and meta‐analysis
Source: Obes Rev. 2025 Apr 3;26(7):e13916. doi: 10.1111/obr.13916 (PMC12137041; doi:10.1111/obr.13916)

Manuscript Title: How do Glucagon-Like Peptide-1 Receptor Agonists Affect Measures of Muscle Mass in Individuals with, and without, Type 2 Diabetes: A Systematic Review and Meta-Analysis

Authors

[1,2]Anyiam, Oluwaseun; [1,2]Ardavani, Arash; [3]Abdul Rashid, Rushdina Sofia; [4]Panesar, Avinash; [1,2]Idris, Iskandar

1. MRC/ARUK Centre for Musculoskeletal Ageing Research and National Institute for Health Research (NIHR), Nottingham Biomedical Research Centre (BRC), School of Medicine, University of Nottingham, Derby, UK.
2. Department of Endocrinology and Diabetes, University Hospitals Derby and Burton NHS Foundation Trust, Derby, UK.
3. Department of Endocrinology and Diabetes, United Lincolnshire Hospitals NHS Trust, Lincoln, UK
4. Derbyshire Healthcare NHS Foundation Trust, Derby, UK

Short Title: GLP1RA effects on muscle mass

Address for correspondence:

Professor Iskandar Idris
University of Nottingham Medical School,
Royal Derby Hospital,
Uttoxeter Road, Derby,
DE22 3DT, UK.

Email: iskandar.idris@nottingham.ac.uk

Appendix S1: PRISMA Checklist

Section and Topic	Item #	Checklist item	Location where item is reported
**TITLE**			
Title	1	Identify the report as a systematic review.	Page 1
**ABSTRACT**			
Abstract	2	See the PRISMA 2020 for Abstracts checklist.	Page 2
**INTRODUCTION**			
Rationale	3	Describe the rationale for the review in the context of existing knowledge.	Page 3
Objectives	4	Provide an explicit statement of the objective(s) or question(s) the review addresses.	Page 3
**METHODS**			
Eligibility criteria	5	Specify the inclusion and exclusion criteria for the review and how studies were grouped for the syntheses.	Page 4
Information sources	6	Specify all databases, registers, websites, organisations, reference lists and other sources searched or consulted to identify studies. Specify the date when each source was last searched or consulted.	Page 4
Search strategy	7	Present the full search strategies for all databases, registers and websites, including any filters and limits used.	Appendix S2-S5
Selection process	8	Specify the methods used to decide whether a study met the inclusion criteria of the review, including how many reviewers screened each record and each report retrieved, whether they worked independently, and if applicable, details of automation tools used in the process.	Page 5
Data collection process	9	Specify the methods used to collect data from reports, including how many reviewers collected data from each report, whether they worked independently, any processes for obtaining or confirming data from study investigators, and if applicable, details of automation tools used in the process.	Page 5
Data items	10a	List and define all outcomes for which data were sought. Specify whether all results that were compatible with each outcome domain in each study were sought (e.g. for all measures, time points, analyses), and if not, the methods used to decide which results to collect.	Page 5
	10b	List and define all other variables for which data were sought (e.g. participant and intervention characteristics, funding sources). Describe any assumptions made about any missing or unclear information.	Page 5
Study risk of bias assessment	11	Specify the methods used to assess risk of bias in the included studies, including details of the tool(s) used, how many reviewers assessed each study and whether they worked independently, and if applicable, details of automation tools used in the process.	Page 5
Effect measures	12	Specify for each outcome the effect measure(s) (e.g. risk ratio, mean difference) used in the synthesis or presentation of results.	Page 5
Synthesis methods	13a	Describe the processes used to decide which studies were eligible for each synthesis (e.g. tabulating the study intervention characteristics and comparing against the planned groups for each synthesis (item #5)).	Page 6
	13b	Describe any methods required to prepare the data for presentation or synthesis, such as handling of missing summary statistics, or data conversions.	Page 6
	13c	Describe any methods used to tabulate or visually display results of individual studies and syntheses.	Page 6
	13d	Describe any methods used to synthesize results and provide a rationale for the choice(s). If meta-analysis was performed, describe the model(s), method(s) to identify the presence and extent of statistical heterogeneity, and software package(s) used.	Pages 5-6
	13e	Describe any methods used to explore possible causes of heterogeneity among study results (e.g. subgroup analysis, meta-regression).	Page 6
	13f	Describe any sensitivity analyses conducted to assess robustness of the synthesized results.	Page 6
Reporting bias assessment	14	Describe any methods used to assess risk of bias due to missing results in a synthesis (arising from reporting biases).	Page 5
Certainty assessment	15	Describe any methods used to assess certainty (or confidence) in the body of evidence for an outcome.	Page 5
**RESULTS**			

Section and Topic	Item #	Checklist item	Location where item is reported
Study selection	16a	Describe the results of the search and selection process, from the number of records identified in the search to the number of studies included in the review, ideally using a flow diagram.	Page 7
	16b	Cite studies that might appear to meet the inclusion criteria, but which were excluded, and explain why they were excluded.	N/A
Study characteristics	17	Cite each included study and present its characteristics.	Supplementary Table S1
Risk of bias in studies	18	Present assessments of risk of bias for each included study.	Supplementary Figures S1-S2 & Tables S5 - S6
Results of individual studies	19	For all outcomes, present, for each study: (a) summary statistics for each group (where appropriate) and (b) an effect estimate and its precision (e.g. confidence/credible interval), ideally using structured tables or plots.	Pages 9-13
Results of syntheses	20a	For each synthesis, briefly summarise the characteristics and risk of bias among contributing studies.	Pages 9-15
	20b	Present results of all statistical syntheses conducted. If meta-analysis was done, present for each the summary estimate and its precision (e.g. confidence/credible interval) and measures of statistical heterogeneity. If comparing groups, describe the direction of the effect.	Pages 9-13
	20c	Present results of all investigations of possible causes of heterogeneity among study results.	Pages 9-14
	20d	Present results of all sensitivity analyses conducted to assess the robustness of the synthesized results.	Page 14
Reporting biases	21	Present assessments of risk of bias due to missing results (arising from reporting biases) for each synthesis assessed.	Page 15
Certainty of evidence	22	Present assessments of certainty (or confidence) in the body of evidence for each outcome assessed.	Pages 13-18
**DISCUSSION**			
Discussion	23a	Provide a general interpretation of the results in the context of other evidence.	Pages 16-17
	23b	Discuss any limitations of the evidence included in the review.	Page 18
	23c	Discuss any limitations of the review processes used.	Page 18
	23d	Discuss implications of the results for practice, policy, and future research.	Page 18
**OTHER INFORMATION**			
Registration and protocol	24a	Provide registration information for the review, including register name and registration number, or state that the review was not registered.	Page 4
	24b	Indicate where the review protocol can be accessed, or state that a protocol was not prepared.	Page 4
	24c	Describe and explain any amendments to information provided at registration or in the protocol.	Page 6
Support	25	Describe sources of financial or non-financial support for the review, and the role of the funders or sponsors in the review.	N/A
Competing interests	26	Declare any competing interests of review authors.	N/A
Availability of data, code and other materials	27	Report which of the following are publicly available and where they can be found: template data collection forms; data extracted from included studies; data used for all analyses; analytic code; any other materials used in the review.	Page 6

Appendix S2: Protocol for Systematic Review / Meta-Analysis

**Title of Review**	The Effect of Glucagon-Like Peptide-1 Receptor Agonists (GLP1RAs) on Lean Body Mass in Overweight Individuals, with and without Type 2 Diabetes: A Systematic Review & Meta-Analysis
**Reviewers**	Dr Oluwaseun Anyiam<br>Dr Arash Ardavani<br>Dr Avinash Panesar<br>Dr Sofia Rashid
**Project Supervisor**	Professor Iskandar Idris

1. Background to review

GLP1RAs are medications that stimulate the receptor of the glucagon-like peptide-1 (GLP-1) hormone. GLP-1 primarily acts in the pancreas to stimulate insulin secretion in response to food ingestion, giving rise to the incretin response. GLP-1 also slows gastric emptying and acts via central nervous system pathways to induce satiety and reduce food consumption. Thus, GLP1RAs exploit these actions of the GLP-1 hormone to improve glycaemic control and reduce body weight, in individuals with type 2 diabetes (T2D) and are now established medications in the management of the condition. More recently, the powerful weight reducing effects of GLP1RAs have led to their approval as treatments for obesity, without T2D also.

Recently, there has been increasing attention around the loss of lean body mass (LBM) associated with weight loss interventions. LBM primarily comprises of skeletal muscle which has a range of important metabolic and physical functions for the maintenance of optimum health. Indeed, skeletal muscle is the largest organ responsible for postprandial glucose uptake, and a low level LBM is associated with increased insulin resistance. Reduced LBM also predisposes to frailty and is independently linked with increased morbidity and mortality.

LBM naturally declines with age from the fourth decade of life, and there is a recognition that exacerbating this process further with weight loss interventions may be detrimental in the long-term. Individuals with obesity are particularly prone to sarcopenia and frailty, making this of key relevance to this population. Moreover, individuals with T2D experience acceleration of the age-related decline in LBM, making this specific group at particularly high risk of these complications. Excess fat mass (FM) characterises overweight and obesity, and is associated with insulin resistance and T2D.

Thus, interventions that preserve LBM, whilst maximising loss of FM are highly desirable overweight individuals, and particularly for individuals with concurrent T2D. One of the less well-described physiological functions of the GLP-1 hormone is simulation of muscle protein synthesis. This suggests that GLP1RAs may be capable of stimulating weight loss whilst concurrently stimulating muscle protein synthesis, thus preserving LBM. Some studies and reviews have suggested this, however no review has quantitatively assessed this using empirical data.

This systematic review and meta-analysis hopes to address this knowledge gap by quantitatively assessing the effect of GLP1RAs on LBM, relative to changes in FM and total weight (TW).

Aims and Research Questions

Aim

To examine the changes in LBM among overweight individuals, with and without T2D, relative to changes in FM and TW.

Research Questions

1: To what extent do GLP1RAs alter LBM, relative to total body weight and fat mass, in overweight individuals?

2: Is there a difference in extent of LBM alteration in individuals with T2D and individuals without T2D

2. Criteria for studies included in the review	
**i. Population**	<u>Inclusion</u><br>• Overweight or obese individuals (mean BMI ≥ 25kgm2)<br>• Individuals with or without type 2 diabetes (mixed cohorts can also be included)<br>• Mean age ≥40 years
**ii. Intervention**	<u>Inclusion</u><br>• Any GLP-1 receptor agonist<br>• Intervention period of ≥ 6 weeks<br>• Can be given with any co-therapy, except insulin<br>• Must include at least one group in which a GLP-1 receptor agonist is the only study intervention
**iii. Comparison**	Any comparator. Studies with no comparator can also be included.
**iv. Outcomes**	Change in the following parameters, based on pre- and post-intervention measurements.<br>Primary Outcomes:<br>• Lean body mass (LBM) – can also include studies that report other surrogate measures of LBM (e.g. LBM percentage, fat-free mass, skeletal muscle mass)<br>Secondary outcomes:<br>• Fat mass (FM)<br>• Total body mass (TM)
**v. Study Design**	Inclusion: All types of studies reported in a full peer-reviewed article and written in English

3. Criteria for excluding studies not covered in inclusion criteria
Studies involving individuals with type 1 diabetes, or pregnant / neonatal / paediatric / adolescent participants. Study population with a mean age <40 years or mean BMI < 25kg/m^2. Studies involving co-therapy with trial-specific insulin use, any GLP-1 receptor/other receptor co-agonist or intervention period <6 weeks. Abstract-only articles, case reports, conference proceedings

4. Search methods	
**Electronic databases**	PubMed<br>Medline<br>EMBASE<br>CINAHL<br>The Cochrane Central Register of Controlled Trials (CENTRAL)<br>Google Scholar
**Other methods used for identifying relevant articles**	Reference checking with identification of potentially relevant articles from articles already identified from primary search (secondary search)

5. Methods of review	
**Details of methods**	Two main reviewers will screen the titles and abstracts of studies identified from the primary search of databases and will decide whether they should progress to eligibility assessment. Any disagreements will be resolved by a third reviewer. Terminology and search process will be clarified beforehand and inter-observer agreement will be calculated using Cohen's d.

	Once potentially eligible studies are identified, two reviewers will assess the full texts to determine whether they include the outcomes relevant to this review. Again, any disagreements will be resolved by a third reviewer.
**Quality Assessment**	Risk of bias will be assessed using the Cochrane Risk of Bias 2 tool for randomised controlled studies, or the relevant Joanna Briggs Institute Critical Appraisal Checklist for non-randomised studies.
**Data extraction**	Data will be extracted into a Microsoft Excel document and will be performed by one reviewer, with a second cross-checking for accuracy. For the main outcomes (LBM, FM & TM), pre-intervention and post-intervention mean and standard deviation (SD) will be collected. If change from baseline is reported instead of post-intervention means, this will be used to calculate the post-intervention mean and the SD will be assumed to be the same as the pre-intervention value.<br><br>In addition, the following details will be obtained from the included articles: GLP1RA dose(s) and frequency, cohort age, study authors, publication date, study design, comparator dose (if applicable), comparator group details (if applicable), main study outcome measures, other outcome measures, data reporting format, and the study-specific findings for the synthesis stage papers.
**Meta-analysis**	A meta-analysis will be performed outlining the absolute changes in LBM, FM and TM reported in identified studies. These will be displayed as individual forest-plots, sub-grouped by diabetes status and GLP1RA dose.<br><br>Heterogeneity will be assessed and further sensitivity analysis will be conducted to determine whether the different methods used to measure body composition or study type have any significant effects on the results.
**Narrative synthesis**	Narrative synthesis will be performed alongside the meta-analysis. This will describe the results of the meta-analysis, relating changes in LBM to changes in FM and TM. This will also be discussed in the context of individuals with and without T2D. Studies that could not be included in the meta-analysis will be discussed in the narrative synthesis also (if applicable), and an assessment of the robustness of the findings will also be performed.

**6. Presentation of results**	
**Additional material**	Protocol<br>Flow chart of the search process<br>Search strategy<br>Data extraction table
**Outputs from review**	Manuscript to be submitted to a high quality journal in the field of obesity or diabetes

**7. Timeline for review**	
**Protocol**	2 weeks
**Literature searching**	2 weeks
**Eligibility assessment**	1 month
**Data extraction & synthesis**	1 month
**Writing up**	1 month

Appendix S3: Medline search strategy

Database:
Ovid MEDLINE(R) ALL <1946 to November 14, 2023>

#	Query	Results from 15 Nov 2023
1	exp Body Weight/	537,229
2	exp Obesity/	264,690
3	exp Diabetes Mellitus/	514,470
4	exp Glucagon-Like Peptide 1/	11,248
5	glp1 receptor agonist.mp. [mp=title, book title, abstract, original title, name of substance word, subject heading word, floating sub-heading word, keyword heading word, organism supplementary concept word, protocol supplementary concept word, rare disease supplementary concept word, unique identifier, synonyms, population supplementary concept word, anatomy supplementary concept word]	48
6	glp-1 receptor agonist.mp. [mp=title, book title, abstract, original title, name of substance word, subject heading word, floating sub-heading word, keyword heading word, organism supplementary concept word, protocol supplementary concept word, rare disease supplementary concept word, unique identifier, synonyms, population supplementary concept word, anatomy supplementary concept word]	1,587
7	Incretins/	2,430
8	incretin mimetic.mp.	170
9	liraglutide.mp.	4,189
10	semaglutide.mp.	1,388
11	exp Exenatide/	2,918
12	dulaglutide.mp.	759
13	lixisenatide.mp. [mp=title, book title, abstract, original title, name of substance word, subject heading word, floating sub-heading word, keyword heading word, organism supplementary concept word, protocol supplementary concept word, rare disease supplementary concept word, unique identifier, synonyms, population supplementary concept word, anatomy supplementary concept word]	596
14	lixisenatide.mp.	596
15	albiglutide.mp.	244
16	glp1ra.mp.	103
17	exp Body Composition/	64,355
18	"fat free mass".mp.	9,588
19	"muscle mass".mp.	26,276
20	"fat mass".mp.	26,937
21	"lean body mass".mp.	8,811
22	lbm.mp.	2,596
23	fm.mp.	25,103
24	body composition.mp. [mp=title, book title, abstract, original title, name of substance word, subject heading word, floating sub-heading word, keyword heading word, organism supplementary concept word, protocol supplementary concept word, rare disease supplementary concept word, unique identifier, synonyms, population supplementary concept word, anatomy supplementary concept word]	71,148
25	obes*.mp. [mp=title, book title, abstract, original title, name of substance word, subject heading word, floating sub-heading word, keyword heading word, organism supplementary concept word, protocol supplementary concept word, rare disease supplementary concept word, unique identifier, synonyms, population supplementary concept word, anatomy supplementary concept word]	450,423
26	type 2 diabetes.mp. [mp=title, book title, abstract, original title, name of substance word, subject heading word, floating sub-heading word, keyword heading word, organism supplementary concept word, protocol supplementary concept word, rare disease supplementary concept word, unique identifier, synonyms, population supplementary concept word, anatomy supplementary concept word]	171,738

27	overweight.mp. [mp=title, book title, abstract, original title, name of substance word, subject heading word, floating sub-heading word, keyword heading word, organism supplementary concept word, protocol supplementary concept word, rare disease supplementary concept word, unique identifier, synonyms, population supplementary concept word, anatomy supplementary concept word]	95,316
28	"body fat".mp. [mp=title, book title, abstract, original title, name of substance word, subject heading word, floating sub-heading word, keyword heading word, organism supplementary concept word, protocol supplementary concept word, rare disease supplementary concept word, unique identifier, synonyms, population supplementary concept word, anatomy supplementary concept word]	39,897
29	1 or 2 or 3 or 25 or 26 or 27	1,193,172
30	exenatide.mp. [mp=title, book title, abstract, original title, name of substance word, subject heading word, floating sub-heading word, keyword heading word, organism supplementary concept word, protocol supplementary concept word, rare disease supplementary concept word, unique identifier, synonyms, population supplementary concept word, anatomy supplementary concept word]	3,868
31	"fat distribution".mp. [mp=title, book title, abstract, original title, name of substance word, subject heading word, floating sub-heading word, keyword heading word, organism supplementary concept word, protocol supplementary concept word, rare disease supplementary concept word, unique identifier, synonyms, population supplementary concept word, anatomy supplementary concept word]	8,577
32	17 or 18 or 19 or 20 or 21 or 22 or 23 or 24 or 28 or 31	159,304
33	4 or 5 or 6 or 7 or 8 or 9 or 10 or 11 or 12 or 13 or 14 or 15 or 16 or 30	17,822
34	29 and 32 and 33	488

Appendix S4: Pubmed search strategy

((((((muscle mass[Title/Abstract]) OR (fat mass[Title/Abstract])) OR (fat free mass[Title/Abstract])) OR (lean body mass[Title/Abstract])) OR (body composition[Title/Abstract])) AND ((((((((albiglutide[Title/Abstract]) OR (lixisenatide[Title/Abstract])) OR (dulaglutide[Title/Abstract])) OR (exen*[Title/Abstract])) OR (semaglutide[Title/Abstract])) OR (liraglutide[Title/Abstract])) OR (GLP1RA[Title/Abstract])) OR (glucagon like peptide 1 receptor agonist[Title/Abstract]))) AND (((overweight[Title/Abstract]) OR (diabet*[Title/Abstract])) OR (obes*[Title/Abstract]))

Appendix S5: EMBASE search strategy

Database:
Embase <1974 to 2023 November 14>

#	Query	Results from 15 Nov 2023
1	obes*.mp. [mp=title, abstract, heading word, drug trade name, original title, device manufacturer, drug manufacturer, device trade name, keyword heading word, floating subheading word, candidate term word]	750,904
2	exp obesity/	672,765
3	exp diabetic obesity/	4,728
4	exp diabetes mellitus/	1,240,805
5	diabet*.mp. [mp=title, abstract, heading word, drug trade name, original title, device manufacturer, drug manufacturer, device trade name, keyword heading word, floating subheading word, candidate term word]	1,473,875
6	overweight.mp. [mp=title, abstract, heading word, drug trade name, original title, device manufacturer, drug manufacturer, device trade name, keyword heading word, floating subheading word, candidate term word]	137,173
7	1 or 2 or 3 or 4 or 5 or 6	2,023,063
8	exp glucagon like peptide 1 receptor agonist/	49,574
9	liraglutide.mp. [mp=title, abstract, heading word, drug trade name, original title, device manufacturer, drug manufacturer, device trade name, keyword heading word, floating subheading word, candidate term word]	13,505
10	semaglutide.mp. [mp=title, abstract, heading word, drug trade name, original title, device manufacturer, drug manufacturer, device trade name, keyword heading word, floating subheading word, candidate term word]	4,302
11	dulaglutide.mp. [mp=title, abstract, heading word, drug trade name, original title, device manufacturer, drug manufacturer, device trade name, keyword heading word, floating subheading word, candidate term word]	2,994
12	lixisenatide.mp. [mp=title, abstract, heading word, drug trade name, original title, device manufacturer, drug manufacturer, device trade name, keyword heading word, floating subheading word, candidate term word]	2,393
13	albiglutide.mp. [mp=title, abstract, heading word, drug trade name, original title, device manufacturer, drug manufacturer, device trade name, keyword heading word, floating subheading word, candidate term word]	1,453
14	exenatide.mp. [mp=title, abstract, heading word, drug trade name, original title, device manufacturer, drug manufacturer, device trade name, keyword heading word, floating subheading word, candidate term word]	4,623
15	8 or 9 or 10 or 11 or 12 or 13 or 14	50,039
16	lean body mass.ti.	986
17	lean body mass.ti,ab.	11,962
18	fat mass.ti,ab.	39,020
19	fat free mass.ti,ab.	12,764
20	muscle mass.ti,ab.	37,467
21	body composition.ti,ab.	63,784
22	16 or 17 or 18 or 19 or 20 or 21	122,358
23	7 and 15 and 22	877

Table S1: Sensitivity analysis – results from studies reporting full data set only

		Weighted Mean Difference	95% confidence interval	p-value
**T2DM LBM:FM comparison**	**LBM**	-0.58kg	-1.59 to 0.44	0.27
	**FM**	-3.12kg	-4.23 to -2.02	<0.0001
**T2DM LBM:Weight comparison**	**LBM**	-0.62kg	-1.70 to 0.46	0.26
	**Weight**	-4.27kg	-6.08 to -2.46	<0.0001
**Non-DM LBM:FM comparison**	**LBM**	-1.31kg	-2.51 to -0.12	0.03
	**FM**	-7.80kg	-10.55 to -5.04	<0.0001
**Non-DM LBM:Weight comparison**	**LBM**	-1.33kg	-2.55 to -0.10	0.03
	**Weight**	-11.38kg	-15.29 to -7.48	<0.0001

Table S2: Sensitivity analysis – results from RCT studies only

		Weighted Mean Difference	95% confidence interval	p-value
**T2DM LBM:FM comparison**	**LBM**	-0.95kg	-2.45 to 0.54	0.21
	**FM**	-2.85kg	-4.14 to -1.55	<0.0001
**T2DM LBM:Weight comparison**	**LBM**	-1.02kg	-2.48 to 0.43	0.17
	**Weight**	-4.23kg	-6.27 to -2.19	<0.0001
**Non-DM LBM:FM comparison**	**LBM**	-1.24kg	-2.32 to -0.16	0.02
	**FM**	-5.45kg	-7.01 to -3.89	<0.0001
**Non-DM LBM:Weight comparison**	**LBM**	-0.51kg	-1.71 to 0.69	0.40
	**Weight**	-3.63kg	-9.51 to 2.24	0.23

Table S3: Sensitivity analysis – result by method of body composition assessment

			Weighted Mean Difference	95% confidence interval	p-value
T2DM	LBM	ADP	-0.63kg	-4.39 to 3.12	0.74
		BIA	-0.56kg	-1.69 to 0.57	0.33
		DXA	-1.06kg	-2.56 to 0.44	0.16
			**Test of group differences**		**0.87**
	FM	ADP	-5.26kg	-8.25 to -2.27	0.0006
		BIA	-3.26kg	-4.60 to -1.91	<0.0001
		DXA	-2.69kg	-4.03 to -1.35	<0.0001
			**Test of group differences**		**0.30**
Non-DM	LBM	BIA	-1.51kg	-2.48 to -0.54	0.002
		DXA	-3.22kg	-5.16 to -1.28	0.001
			**Test of group differences**		**0.12**
	FM	BIA	-7.27kg	-11.09 to -3.44	0.0002
		DXA	-6.60kg	-8.11 to -4.89	<0.0001
			**Test of group differences**		**0.75**

Table S4: Sensitivity analysis – result by measure of SM mass

		Weighted Mean Difference	95% confidence interval	p-value
T2DM	FFM	-0.99kg	-2.32 to 0.34	0.14
	LBM	-1.00kg	-2.43 to 0.43	0.17
	SMM	-0.39kg	-1.63 to 2.41	0.71
		**Test of group differences**		**0.48**
Non-DM	LBM	-2.96kg	-4.77 to -1.15	0.001
	SMM	-1.51kg	-2.49 to -0.54	0.002
		**Test of group differences**		**0.17**

Table S5: Sensitivity analysis – subcutaneous v oral Semaglutide

		Weighted Mean Difference	95% confidence interval	p-value
MM	Subcutaneous	-1.80kg	-3.61 to 0.01	0.05
	Oral	-0.60kg	-2.41 to 3.61	0.69
		**Test of group differences**		**0.18**
FM	Subcutaneous	-5.17kg	-7.26 to -3.08	<0.0001
	Oral	-4.08kg	-6.53 to -1.64	0.001
		**Test of group differences**		**0.51**

Figure S1: Risk of bias assessment results for RCT studies

	Risk of bias domains					
**Study**	**D1**	**D2**	**D3**	**D4**	**D5**	**Overall**
Harder *et al.*, 2004	+	+	+	+	!	!
Jendle *et al.*, 2009	+	+	+	+	!	!
Bunck *et al.*, 2010	!	+	+	+	!	!
Astrup *et al.*, 2012	+	+	+	+	!	!
Yin *et al.*, 2018	+	+	+	!	+	!
Feng *et al.*, 2019	+	+	+	!	!	!
van Eyk *et al.*, 2019	+	+	+	+	+	+
Kadouh *et al.*, 2020	+	+	+	+	!	!
McCrimmon *et al.*, 2020	+	+	+	+	+	+
Lundgren *et al.*, 2021	+	+	+	+	+	+
Neeland *et al.*, 2021	+	+	+	+	+	+
Wilding *et al.*, 2021	+	+	+	+	+	+
Yabe *et al.*, 2022	+	+	+	+	!	!
Heise *et al.*, 2023	+	+	+	+	!	!
Silver *et al.*, 2023	+	+	+	+	!	!

Figure S2: Risk of bias assessment results for Crossover RCT studies

	Risk of bias domains						
**Study**	**D1**	**DS**	**D2**	**D3**	**D4**	**D5**	**Overall**
Blundell *et al.*, 2017	!	+	+	+	+	!	!
Gibbons *et al.*, 2021	!	+	+	+	+	!	!

Table S6: Risk of bias assessments for single group non-RCT studies

	Diaz-Soto *et al.,* 2013	Li *et al.,* 2014	Hong *et al.,* 2016	Perna *et al.,* 2016	Rondanelli *et al.,* 2016	Seko *et al.,* 2017	Ishii *et al.,* 2019	Park *et al.,* 2021	Ozeki *et al.,* 2022	Volpe, Lisco, Fanelli *et al.,* 2022	Volpe, Lisco, Racaniello *et al.,* 2022	Santini *et al.,* 2023	Uchiyama *et al.,* 2023	Volpe *et al.,* 2023
**Were there clear criteria for inclusion in the case series?**	N	Y	N	Y	Y	Y	Y	Y	N	Y	Y	Y	Y	Y
**Was the condition measured in a standard, reliable way for all participants included in the case series?**	Y	Y	Y	Y	Y	Y	Y	Y	Y	Y	Y	Y	Y	Y
**Were valid methods used for identification of the condition for all participants included in the case series?**	Y	Y	Y	Y	Y	Y	Y	Y	Y	Y	Y	Y	Y	Y
**Did the case series have consecutive inclusion of participants?**	Y	Y	U	Y	Y	Y	Y	Y	Y	Y	Y	Y	U	Y
**Did the case series have complete inclusion of participants?**	Y	Y	U	Y	Y	Y	Y	Y	Y	Y	Y	Y	U	Y
**Was there clear reporting of the demographics of the participants in the study?**	Y	Y	N	Y	Y	Y	Y	Y	N	Y	Y	Y	Y	Y
**Was there clear reporting of clinical information of the participants?**	Y	Y	Y	Y	Y	Y	Y	Y	Y	Y	Y	Y	Y	Y
**Were the outcomes or follow up results of cases clearly reported?**	Y	Y	Y	Y	Y	Y	Y	Y	Y	Y	Y	Y	Y	Y
**Was there clear reporting of the presenting site(s)/clinic(s) demographic information?**	Y	Y	N	Y	Y	Y	Y	Y	Y	Y	Y	Y	Y	Y
**Was statistical analysis appropriate?**	Y	Y	Y	N	Y	U	Y	Y	Y	Y	Y	Y	Y	Y

Table S7: Risk of bias assessment for multiple group non-RCT studies

	de Luis et al., 2014	de Luis et al., 2015	Timofte et al., 2015	Iepsen et al., 2018	Agcakaya et al. 2023	Frietas et al., 2023	Stefanakis et al., 2023
**Were the two groups similar and recruited from the same population?**	Y	Y	Y	N/A	Y	Y	Y
**Were the exposures measured similarly to assign people to both exposed and unexposed groups?**	N/A	N/A	N/A	N/A	N/A	N/A	N/A
**Was the exposure measured in a valid and reliable way?**	N/A	N/A	N/A	N/A	N/A	N/A	N/A
**Were confounding factors identified?**	Y	Y	N	Y	Y	Y	Y
**Were strategies to deal with confounding factors stated?**	N	Y	N	Y	Y	Y	Y
**Were the groups/participants free of the outcome at the start of the study (or at the moment of exposure)?**	N/A	N/A	N/A	N/A	N/A	N/A	N/A
**Were the outcomes measured in a valid and reliable way?**	Y	Y	Y	Y	Y	Y	Y
**Was the follow up time reported and sufficient to be long enough for outcomes to occur?**	Y	Y	Y	Y	Y	Y	Y
**Was follow up complete, and if not, were the reasons to loss to follow up described and explored?**	Y	Y	Y	Y	Y	Y	Y
**Were strategies to address incomplete follow up utilized?**	N/A	N/A	N/A	N/A	N	N/A	N/A
**Was statistical analysis appropriate?**	Y	Y	Y	Y	Y	Y	Y

Figure S3: Funnel plot of T2DM studies reporting measures of MM

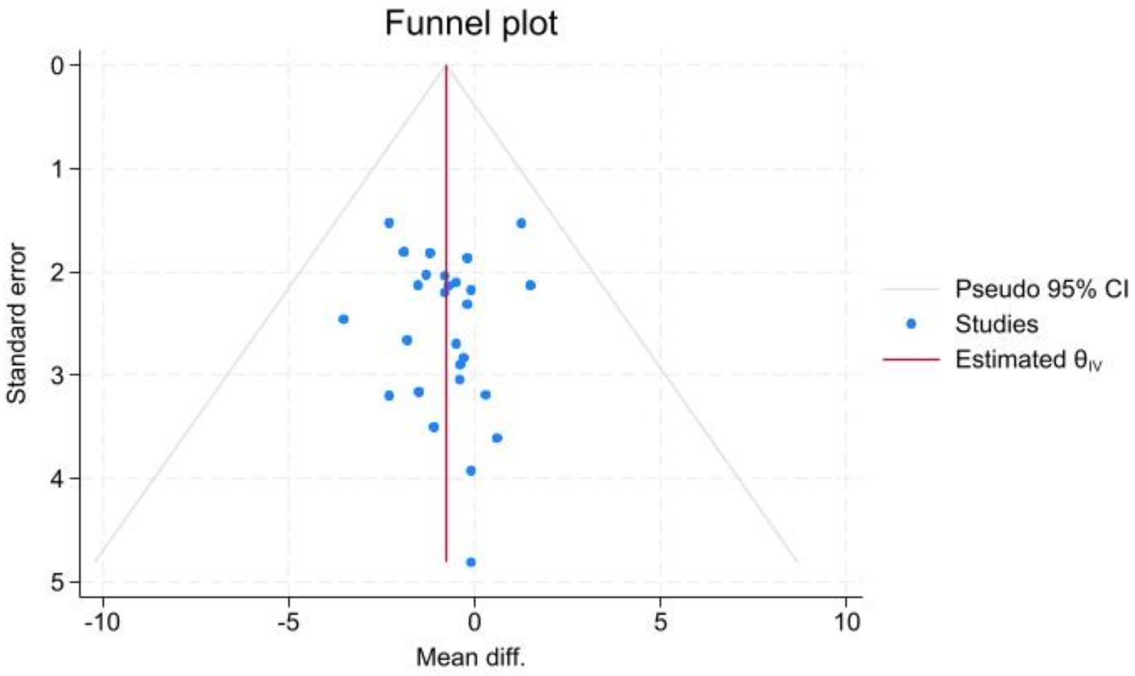

Figure S4: Funnel plot of non-DM studies reporting measures of MM

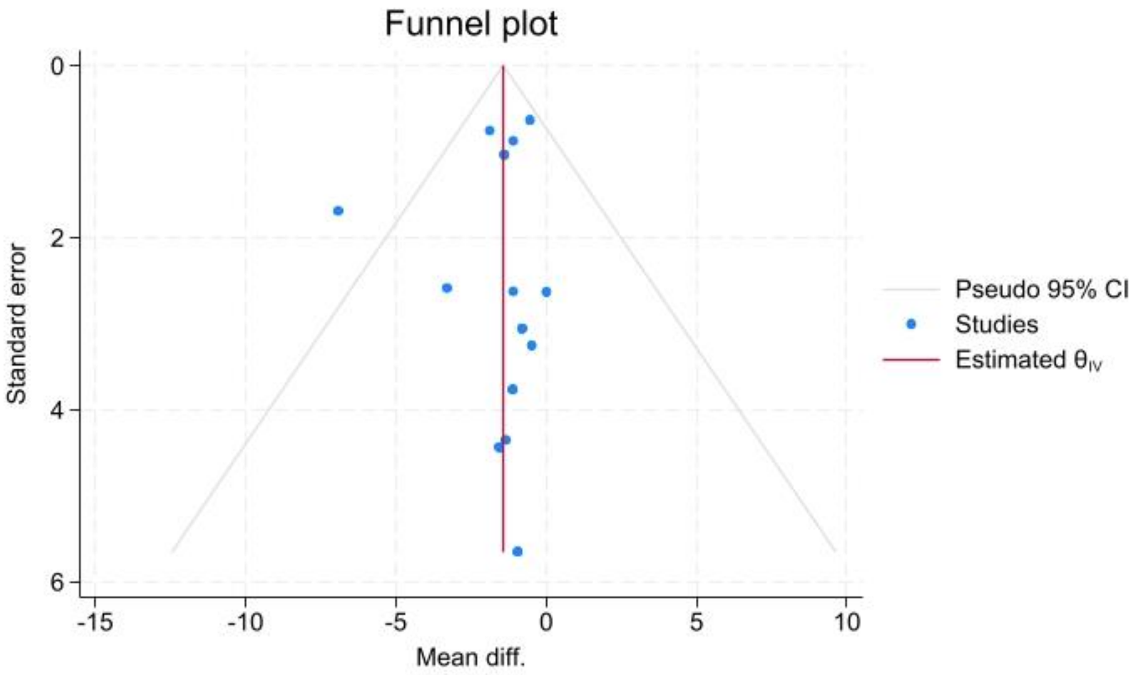

Supplement: Supplementary file 1 — Appendix S1: PRISMA Checklist Appendix S2: Protocol for Systematic Review/Meta‐Analysis Appendix S3: Medline search strategy Appendix S4: Pubmed search strategy Appendix S5: EMBASE search strategy Table S1: Sensitivity analysis – results from studies reporting full data set only Table S2: Sensitivity analysis – results from RCT studies only Table S3: Sensitivity analysis – result by method of body composition assessment Table S4: Sensitivity analysis – result by measure of SM mass Table S5: Sensitivity analysis – subcutaneous v oral Semaglutide Figure S1: Risk of bias assessment results for RCT studies Figure S2: Risk of bias assessment results for Crossover RCT studies Table S6: Risk of bias assessments for single group non‐RCT studies. Table S7: Risk of bias assessment for multiple group non‐RCT studies Figure S3: Funnel plot of T2DM studies reporting measures of MM Figure S4: Funnel plot of non‐DM studies reporting measures of MM [file OBR-26-e13916-s001.pdf]
